# Computer-Aided Multi-Target Management of Emergent Alzheimer's Disease

**DOI:** 10.6026/97320630014167

**Published:** 2018-05-05

**Authors:** Hyunjo Kim, Hyunwook Han

**Affiliations:** 1 Department of Medical Informatics, Ajou Medical University Hospital, Suwon, Kyeounggido province, South Korea; 2 Department of Informatics, School of Medicine, CHA University, Seongnam, South Korea; 3 Institute of Basic Medical Sciences, School of Medicine, CHA University, Seongnam, South Korea

**Keywords:** Alzheimer's disease, treatment modeling algorithms, memory complications, chronic neuro-degenerative disorder, dementia prediction, theragnosis

## Abstract

Alzheimer's disease (AD) represents an enormous global health burden in terms of human suffering and economic cost. AD management requires a shift from the prevailing paradigm targeting pathogenesis to design and develop effective drugs with adequate success in clinical trials. Therefore, it is of interest to report a review on amyloid beta (Aβ) effects and other multi-targets including cholinesterase, NFTs, tau protein and TNF associated with brain cell death to be neuro-protective from AD. It should be noted that these molecules have been generated either by target-based or phenotypic methods. Hence, the use of recent advancements in nanomedicine and other natural compounds screening tools as a feasible alternative for circumventing specific liabilities is realized. We review recent developments in the design and identification of neuro-degenerative compounds against AD generated using current advancements in computational multi-target modeling algorithms reflected by theragnosis (combination of diagnostic tests and therapy) concern.

## Background

Drug development for Alzheimer's disease (AD) began with the proposal of the cholinergic hypothesis for memory impairment [1, 2, 3, 4, 5, 6, 7, 8, 9, 10, 11, 12, 13, 14, 15, 16, 17, 18, 19, 20, 21, 22, 23, 24, 25, 26, 27, 28, 29, 30, 31, 32, 33, 34, 35, 36]. There are only four known cholinesterase inhibitors despite the evaluation of numerous potential treatments in clinical trials [7, 11, 14, 23, 25, 29, 33, 34, 35, 37, 38, 39, 40]. The amyloid hypothesis which points to amyloid β-peptide (Aβ) [8, 20, 23, 32, 41, 42, 43, 44, 45, 46] as the initiating factor in AD had a central role in the development of therapeutic strategies on the synapses of analyzing its contribution to AD pathology and discussing its potential as pharmacological target. Alzheimer's disease is also characterized by the presence of tau protein [2, 6, 10, 12, 14, 16, 18, 27, 29, 46, 47, 48, 49, 50] and neuro-fibrillary tangles (NFTs) [1, 12, 14, 45, 46, 50] in the brain as a common neuro-degenerative disorder [1, 2, 6, 11, 23, 28, 29, 30, 36, 37, 43, 45, 51, 52]. Various higher vertebrate models [45] have been used to study the pathophysiology of AD. The criteria extend from prodromal (early) to mild-cognitive impairment of the disease. Early diagnosis plays an important role in preventing progress [41, 47, 53] or lateonset of AD. Treatment for AD is based on features in brain image [54, 55, 56, 57, 58, 59, 60, 61, 61, 62, 63, 64, 65]. The features include AD-related variations of anatomical brain structures such as ventricles size, hippocampus shape, cortical thickness, and brain volume. Prediction of AD is possible with a deep 3D convolution neural network (3D-CNN) [56] which learns generic features capturing AD biomarkers and adjusts to different domain datasets. Characteristics such as cognitive performance, activities of daily living, global change and severity ratings have persisted as the primary clinically relevant outcomes.

Regulatory guidance has helped in the enrichment of early-stage AD trial samples by using biomarkers [25, 28, 47, 56, 66] and phase-specific outcomes. We believe that the model of "one disease - one assay - one drug" is applicable to AD which is one of the most common neuro-degenerative diseases. The discrete complexities in the molecular pathogenesis combined with limited knowledge on the inherited and sporadic forms of the disease together the heterogeneity in the clinical development despite the surplus in available yet validated biomarkers for early diagnosis or prognosis of AD has been established [67, 68, 69, 70, 71, 72, 73]. Thus, a different way of thinking is in demand for a comprehensive explanation of the molecular pathogenesis of the disease. Therefore, it is of interest to review the recent advancements in systems biology towards a complete understanding of AD mechanisms emphasizing the emergence of various highthroughput strategies for improvement drug development using OMICS data.

### Computational modeling analysis of AD targets

We reviewed data on late-stage drug development for AD over the 4 decades [3, 25]. Drug-like molecules with cholinergic function with modest and consistent clinical effects in late-phase trials are known. Hence, there is a need for further improvement in the development of AD specific drugs. Data is also available on late clinical development, methods, biomarkers and regulatory issues at the multi functional point of view [74, 75, 76, 77, 78, 79, 80, 81] with the comparison to other neuro-degenerative disorders such as PD for the purpose of neuro-protective effects [82, 83, 84]. It should be noted that predominant drug targets are in the cholinergic system and the amyloid cascade although a large range of small molecules and biological products have been investigated in clinical trials. Therefore, there is a need to review and document the available computational methods encompassing ligand-based approaches (QSAR, pharmacophores), structure-based approaches (homology modeling, docking, molecular dynamics simulation), and combined approaches (virtual screening) used in the development of drugs for AD. It is also important to document the comprehensive information related to the molecular etiologies of the disease, novel targets for drug development, and different chemo-informatics modeling strategies in this context. We also document information on multi-target drug development, natural products, protein/peptide biomedicine, natural products, and nano-materials are also included in connection with computational modeling of anti-Alzheimer drug development.

### Data mining in known literature databases for AD

We used the available literature databases for gathering information related to AD and its drug development. The Pubmed (http://www.ncbi.nlm.nih.gov/pubmed/) database search was completed using keywords from 01/January/2008 until 31/January/2018 for literature data on AD. Keywords such as "Alzheimer's disease", "memory complications", "chronic neurodegenerative", "dementia prediction", "theragnostics", and "treatment algorithms" were used. "Alzheimer's disease" and "treatment algorithms" produced 3844 abstracts. This data was further manually curated for knowledge enchainment. We also used the http://www. clinicaltrials.gov/ database for AD related clinical data.

### IRP data related to AD

Intellectual property rights (IPR) data related to AD is highly relevant in drug discovery. We gathered IRP data related to AD made available from 1997 to 2017 using KIPRIS (Korean patents (KP)), WISDOMAIN (worldwide patents), IPIntellisource (USP, EP, JP (PAJ), CN (China) and PCT).

### AD related patent data

**Figure 1:**
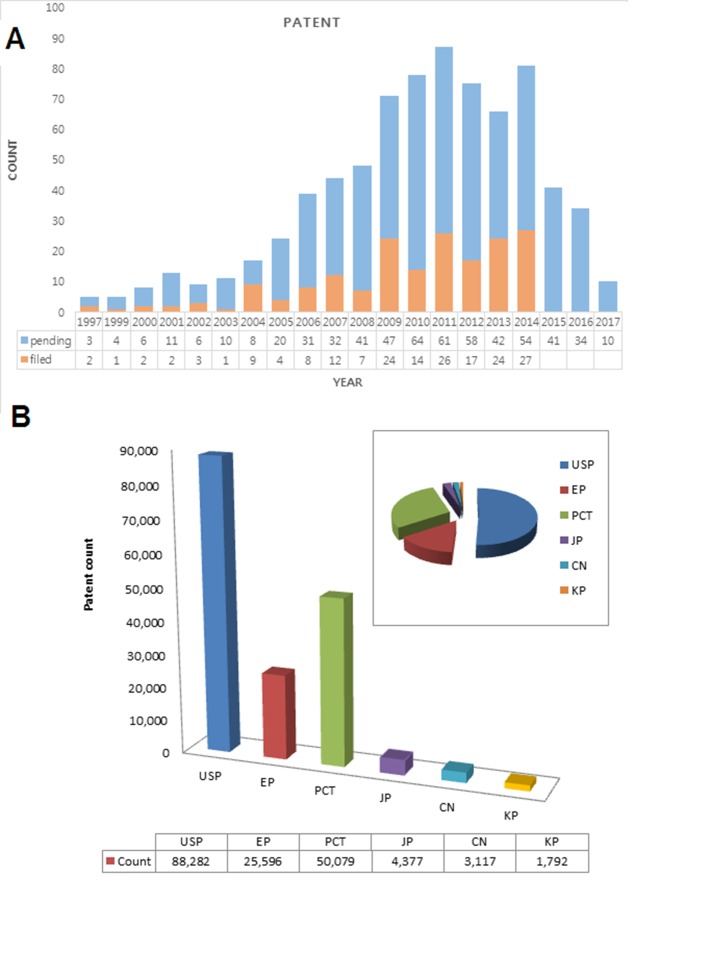
(A) Distribution of patentsfiled for AD related drugs from 1997 to 2017; (B) Distribution of patents filed for AD related drugs on the basis of patent offices across the world.

The treatment of AD poses perplexing challenges due to the complex pathology involved in the etiology of the disease. Drugdiscovery have to shift focus from the design of selective agents that target only one patho-physiological pathway to the design of agents that operate through manifold mechanisms targeting the complexity of the disease state. Patent analysis on new drugs for AD shows that the trend on patent submission has increased remarkably since 2005 (Figure 1a). The continuous clinical trial failures require a shift from the prevailing paradigm targeting pathogenesis to the multi functional one. AD is emerging as the most prevalent and socially disruptive illness of aging populations. Therefore, manifold targeting using a combination of drug entities has been used in the clinical setting for several years through a poly pharmacy approach. This poly pharmacy has been achieved by combining several drugs that independently act on different etiological targets of the disease. Moreover, it should that about 50% patents filed are held by companies in USA (Figure 1b). Furthermore, localized delivery by means of nano medicines limiting the side effects of anti-AD agents should be effective at improving AD management. However, some important concerns were to be addressed in this regard. Clinical efficacy and potential toxicity of naturally available active compounds in large trials also require further assessment before their use in clinical practice.

### Computer-aided mathematical model for AD

It is known that a mathematical model for AD consists of neurons, astrocytes, microglia, and peripheral macrophages as well as Aβ aggregation and hyper-phosphorylated tau proteins. This model is described by a system of partial differential equations. This model is used to simulate the effect of drugs that are either failed in clinical trials, or currently in clinical trials. These simulations suggest that a combined therapy with TNF-α inhibitor and anti-Aβ could yield significant efficacy in slowing the progression of AD [85, 86, 87, 88].

### Equations for Aβ

The Aβ within neurons, Aiβ is constitutively released from amyloid precursor protein (APP) at a rate,λiβ and it is degraded at a rate, Aiβ. Aiβ is overproduced under the reactive oxidative stress (ROS) designated as R.

Hence the equation for is given by

∂Aiβ/∂t = (λiβ (1+R) - (dAiβ . Aiβ ) N/N0 - [1]

Where, N0 is the reference density of the neuron cells in the brain.

### Equation for neurons

Hyper-phosphonated tau proteins forming neuro fibrillary tangles cause microtubules de-polymerization and destruction resulting in neuron death [89, 90, 91, 92, 93, 94, 95, 96]. However, neuron death is also caused by stress from pro-inflammatory cytokines that is resisted by anti-inflammatory cytokines. We represent the proinflammatory cytokines by TNF- α and the anti-inflammatory cytokines by IL-10.

Hence, the equation for N takes the following form:

∂A/∂t= -dNF . Fi/Fi +kFi . N-dNT . Ta/Ta +kTa . 1/1+γ10/kl10 . N - [2]

Where, the death rate of N caused by Fi and Tα are assumed to depend on their saturation levels.

**Figure 2:**
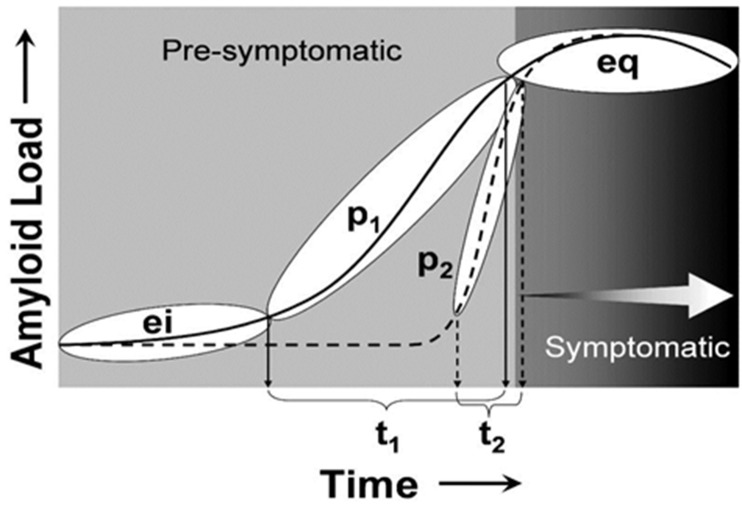
A hypothetical schematic illustrating the progression of amyloid deposition over time from early initiation (ei) phase to progressive phase (p1, p2) leading to the final late equilibrium (eq) phase is shown.

Imaging agents capable of assessing in vivo Aβ content in the brains for AD subjects is important as diagnostic agents to detect Aβ plaques to help test the amyloid cascade hypothesis. This aids to assess the efficacy of anti-amyloid therapeutics under development in clinical trials. The hypothetical schematic of the progression of amyloid deposition over time from the very early initiation (ei) phase to the continuously progressive (p) phase and to final late equilibrium (eq) phase is illustrated. It should be noted that relatively long (p1/t1) and brief (p2/t2) progressive phases as shown in Figure 2. Symptoms are not evident until the equilibrium (eq) phase but the cascade of pathological events that leads to these symptoms (i.e., neuro fibrillary pathology and synapse loss) is initiated during the progressive phase (p).

### Multi-Target designed Ligands (MTDL) against AD

Multiple factors involved in AD include amyloid aggregation to form insoluble neuro toxic plaques of Aβ, hyper-phosphorylation of tau protein, oxidative stress, calcium imbalance, mitochondrial dysfunction, and deterioration of synaptic transmission. These factors together accentuate changes in the CNS homeostasis starting a complex process of interconnected physiological damage leading to cognitive and memory impairment and neuronal death. The rational design of new drug candidates by multi target-directed ligand (MTDL) developed a variety of hybrid compounds acting simultaneously on diverse biological targets has gained increasing attention in recent years. Therefore, it is of interest to review data related to MTDL in the development of candidates specific to the treatment of AD.

### QSAR model for AD

**Figure 3:**
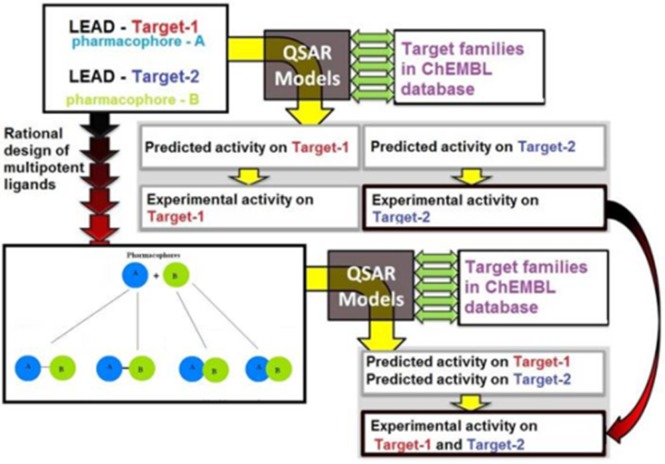
Computer-aided rational design of multi-potent ligands with controlled poly pharmacology is shown using a QSAR model.

QSAR modeling has progressed from analysis of small series of congeners (same kind) using basic regressions to applications on very large and diverse data sets using a variety of statistical and machine learning methods [31]. QSAR uses ligand based theoretical approaches for modeling the physical, biological, and pharmacological properties of compounds and forms a crucial initial step in drug discovery. Combinations of the QSAR approach and related theoretical methods such as virtual screening and docking are very useful in the study and design of multi-target ligands with unique poly-pharmacological profiles (Figure 3). Therefore, the application of QSAR in the identification and design of novel yet effective compounds in the treatment of AD is relevant.

### Chemo-informatics methods for on-Target and off-Target bioactivity prediction

**Figure 4:**
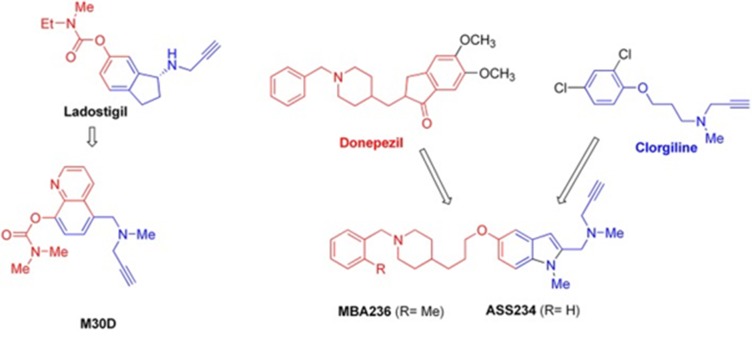
Structures and pharmaco-phores of effective Multi- Target Designed Ligands against AD is shown using a MTDL model.

Multimodal brain permeable drugs affecting a few brain targets involved in the disease pathology such as MAO [97, 98, 99, 100, 101, 102, 103, 104, 105] and ChE enzymes [37, 38, 39, 40], iron accumulation and Aβ generation/aggregation were extensively examined as an essential therapeutic approach in AD treatment. In an example, a hybrid compound contains the key pharmacophores from three drugs such astacrine, rivastigmine (ChEIs), and rasagiline/ladostigil (MAO-B inhibitor) while NCE (New Chemical Entity) contain the pharmacophores of the drugs donepezil (ChEIs) and clorgiline (MAO-A inhibitor). Pharmacophore and 3D-QSAR studies [106, 107, 108, 109] of donepezil and clorgiline derivatives inhibiting both AChE/BuChE and MAOA/ B were successfully applied for lead optimization work and for design of new chemical entities and related ligands with optimal poly-pharmacological and pharmacokinetic profiles. The propargylamine moiety in the MAO-inhibiting pharmacophore of rasagiline, ladostigil or clorgiline is responsible for their neuroprotective and neuro-restorative effects. Thus, propargylamine moiety used as the main chemical scaffold responsible for MAO inhibition in the designed hybrids is illustrated (Figure 4).

### Factorial design of multi target drugs for AD

**Figure 5:**
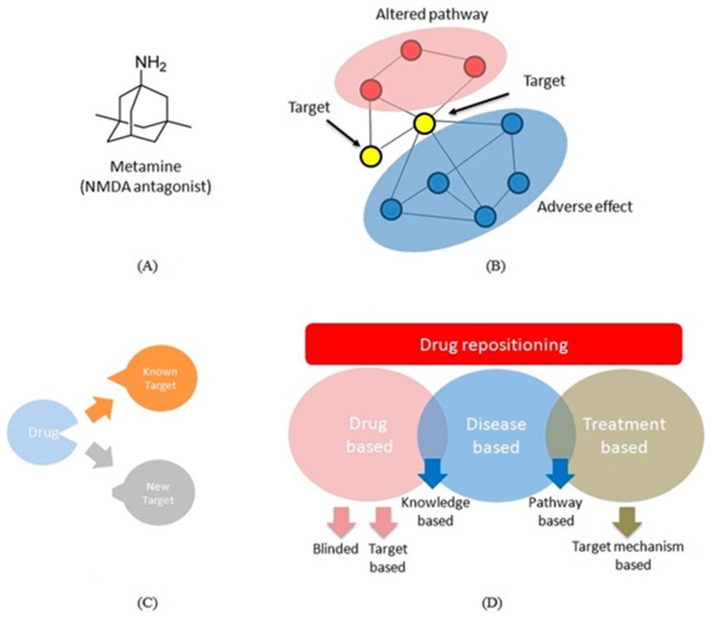
Various techniques such as the chemical structure of (A) metamine; (B) blinded; (C) knowledge based; and the (D) targeted-mechanism based approaches are illustrated using a chemo-informatics model.

Factorial designs of multi target drugs for AD are essential given the enormous and crucial advancements in the knowledge of the mechanisms and implications of AD. Available information on the epigenetics and environment differences specific to AD is crucial in the factorial design of the disease. The NIH National Center for Advancing Translational Sciences (NCATS) maintains NCATS Pharmaceutical Collection database (Table 1). Critical review on this data is highly relevant in this context. NCATS and pharma companies use this database to explore about 3800 known drug compounds using phenotypic data in discovery. Various techniques for repositioning that includes blinded, knowledge-based and targeted-mechanism based as shown in Figure 5 are often used in the design of novel compounds. The chemical structure of Metamine® that is used as a multi-target molecule for AD is illustrated as an example in a chemoinformatics based application of drug design.

### Multi-target-directed ligands (MTDLs) for AD

**Figure 6:**
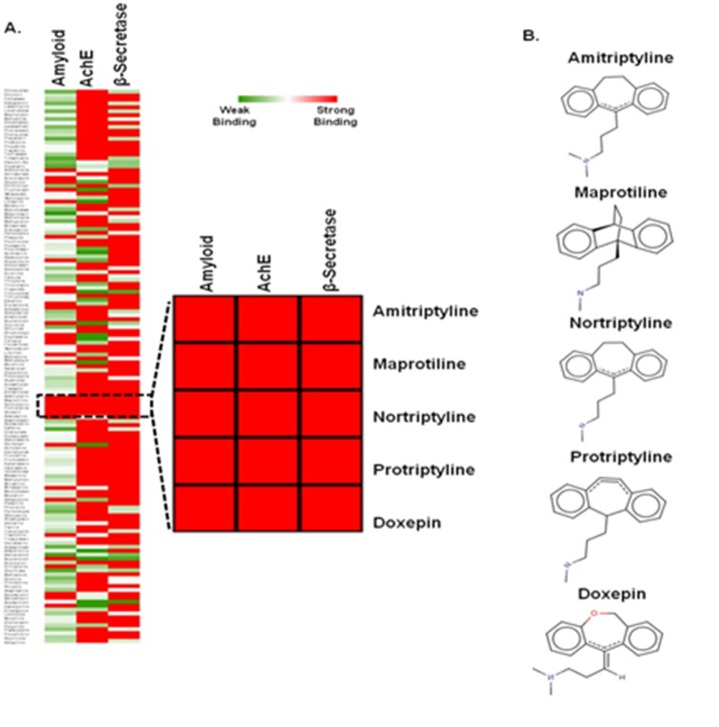
Heat map represented by binding energy scores is shown for several compounds against AD related targets.

Multi-target-directed ligands (MTDLs) [6, 11] offer promising candidates for the treatment of AD. The structures of 140 ligands were docked with the major targets of AD such as AChE, BACE- 1, and Aβ aggregation. Ligands were scored based on electrostatic and hydrophobic contributions to the binding energy. Polar interactions by H-bonding interactions analysis were studied. Docking scores were used to rank ligands depending on presence of number of H-bond donors and acceptors within the active sites. Binding energy scores represented in the Heat map (Figure 6) displayed variability in interactions of the ligands to the three targets of AD. There were several ligands that showed striking interaction with at least two targets and some had strong interaction with all the targets. It was shown that five anti depressant drugs having tricyclic secondary amines had strong binding affinity with broad specificity towards multiple targets of AD. Heat map analysis of binding constants for 140 FDA approved nervous system drugs screened against Aβ, AchE, and β-secretase is also available.

### Virtual screening using molecular docking for AD

**Figure 7:**
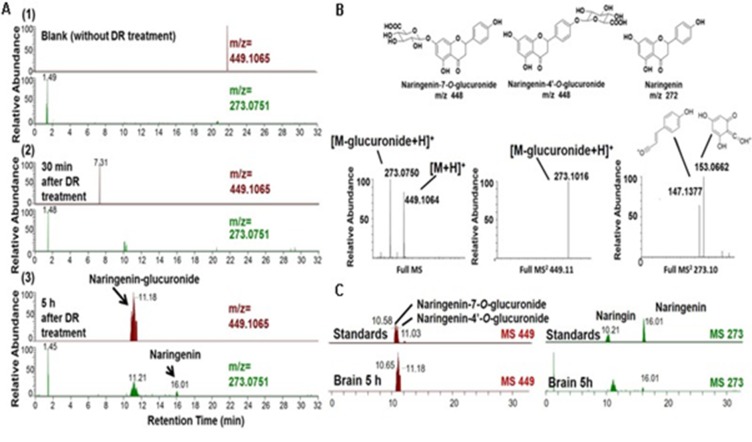
Analysis of naringenin-glucuronide and [M-glucuronide+H]+ used in AD treatment.

Molecular docking [110, 111, 112, 113, 114, 115, 116] enables the extraordinary structural diversity of natural products to be exploited in an efficient manner. The use of molecular docking in virtual screening for the identification of bioactive molecules from natural product databases is feasible. The diversity of chemical components and the unknown bio-metabolism is the challenge in use of natural medicines and the identification of their active constituents. The systematic strategy for evaluating the bioactive candidates in natural medicines used for AD is shown in Figure 7. Beta-site APP cleaving enzyme1 (BACE1) catalyzes [117, 118, 119] the rate determining step in the generation of Aβ peptide and is widely considered as a potential therapeutic target for AD. The active site of BACE1 contains catalytic aspartic (Asp) dyad and flap. Asp dyad cleaves the substrate amyloid precursor protein (APP) with the help of the flap. Available inhibitors against BACE1 are pseudo-peptide or synthetic derivatives. However, there is a need to search for a potent inhibitor with a natural scaffold interacting with the flap and Asp dyad. The natural database InterBioScreen was screened for 3D QSAR pharmacophore modeling, mapping, and ADME/T predictions [120, 121, 122, 123] to find the potential BACE1 inhibitors. Molecular dynamics simulation analysis of the docked compounds provided insights to binding stability. Thus, the use of molecular modeling, docking and simulation is highly relevant in the rational design of potential candidates for AD.

### Ware Drug Discovery Program and Decision tree model for AD

**Figure 8:**
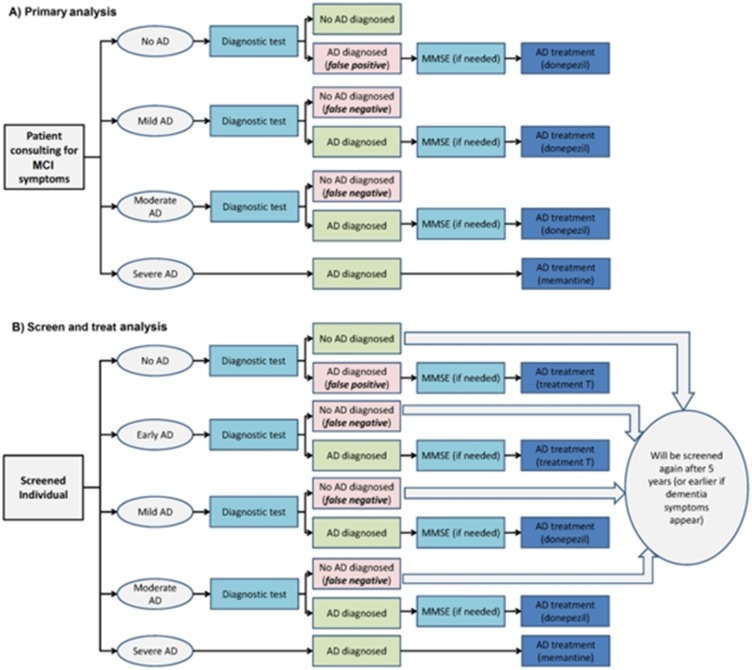
AD treatment scheme is illustrated using possible outcomes of the testing procedure: (a) The primary scenario and (b) The "screen and treat" scenario.

The Ware Drug Discovery Program [38] AD drug extends from target identification to human clinical trials and FDA approval of potential new AD therapies. This method is advanced to the academic centered drug target, biomarker discovery and validation followed by industry driven development of new compounds to clinical trials and FDA approval for marketing. The Ware Alzheimer Drug Discovery Program combines these two critical components into a unified program. The Ware Drug Discovery Program investigates compounds that are not of interest to industry due to lack of IRP issues towards the development of therapies for AD [124, 125, 126, 127, 128, 129, 130]. The generic diagnostic test mentioned in the trees is standard diagnosis; standard MRI or MRI+CLP as shown in Figure 8. The imaging procedures are followed by a cognition test (MMSE) to determine the disease stage when AD is diagnosed. Decision tree was performed and tested for classified patients to administer new molecules for AD treatment as described above.

### Molecular docking analysis for active site inhibitors of MAO-A and B

**Figure 11:**
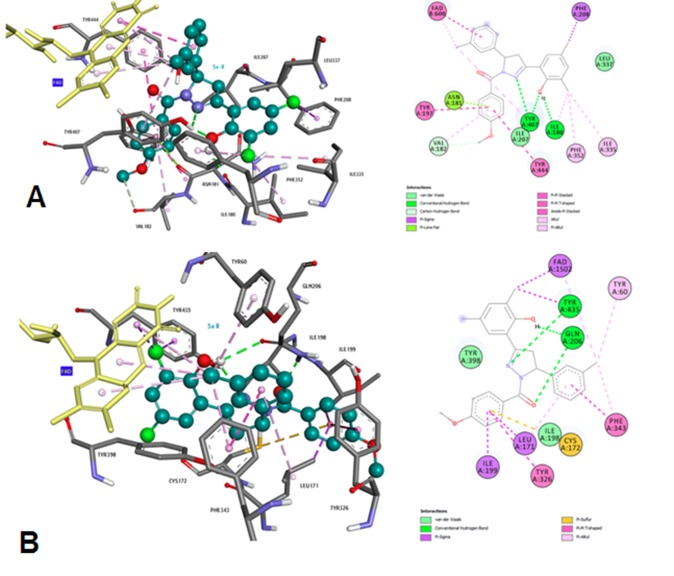
2D/3D representations of compounds binding to the active site of MAO-A in the context of AD are shown.

The use of docking tools in the design of compounds for neurodegenerative diseases is illustrated using MAO-A and B inhibitors using one of the subunits as a target. Water molecules and heteroatoms in the target were removed prior to the docking experiment. Hydrogens were added and the target protein is minimized using the Discovery Studio protocol (accelrys.com) using Chemistry at Harvard Macromolecular Mechanics (CHARMM) force field. Missing hydrogen atoms were added on the basis of the protonation state of the titratable residues. Molecular models of the inhibitors were built and optimized using SPARTAN 10.0 (software for a molecular modelling and computational chemistry application from wave function). Molecular docking was completed as shown in Figure 11 using AutoDock 4.2 (a suite for automated docking of target with ligands). The flavin-N5-oxide atom of the flavin adenine dinucleotide (FAD) molecule, which is a redox cofactor, and more specifically a prosthetic group of a protein involved in several important enzymatic reactions in metabolism cover the entire binding site. Compounds were docked with both MAO-A and MAO-B and the selectivity was compared. Several representative ligands were chosen and the important interactions were visualized in the Accelrys Visualization 4.5 program as shown in Figure 11.

### Theragnosis (combination of diagnostic tests and therapy) for AD

Theragnosis is a new field of medicine, which combines specific targeted therapy based on specific diagnostic tests with a focus on patient centered care. It provides a transition from conventional medicine to a contemporary personalized yet precision medicine. This paradigm involves using nano-science to unite diagnostic and therapeutic applications to form a single agent, allowing for diagnosis, drug delivery and treatment response monitoring. AD presents a pioneering example where research to implement every aspect of predictive, preventive, and personalized medicine is applicable. It should be noted that majority of available biomarkers serve as tools during the investigation of disease progression as well as during novel drug discovery and development.

### TOMM40 variable-length polymorphism and the age of lateonset AD

**Figure 12:**
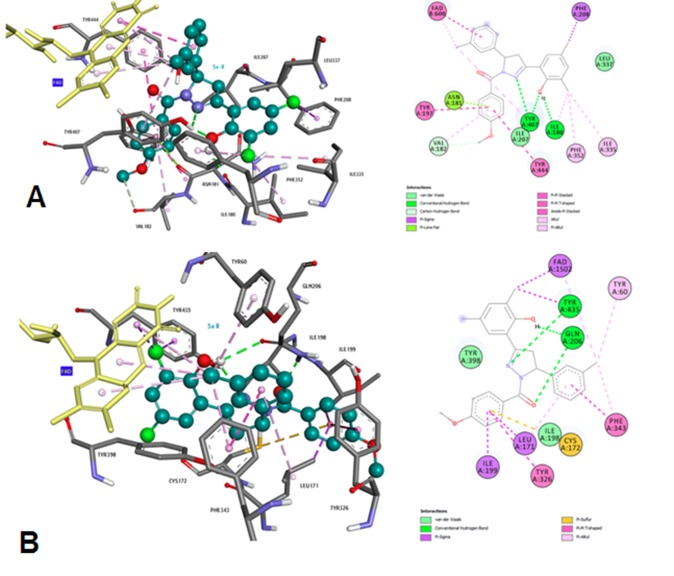
Age of onset for AD to unaffected genotype is shown using TOMM40-APOE haplo-type curve.

Co-localized genetic markers TOMM40 and APOE [141, 142, 143, 144, 145, 146, 147, 148, 149, 150, 151, 151, 153] which account for the vast majority of variability in both risk and age-of-onset of the disease (Figure 12) is useful for the prediction of age of AD onset. It is proposed that each of the original AD age of onset curves is a composite of sub-curves that are defined by TOMM40 genotype. The APOE4/4 curve remains unchanged as the vast majority of APOE4 alleles carry the long TOMM40 allele. There are two curves for APOE3/4 individuals due to the presence of either a shortor a very long polymorphism linked to APOE3. There are three curves for APOE3/3 individuals due to the possible combination of alleles, i.e. short/short (Sh/Sh), short/very long (Sh/VL), and very long/very long (VL/VL). Thus, the commonly accepted assumption that LOAD is underlined by a complex and elaborate set of genetic markers can potentially be countered. The complexity can in fact be disentangled and reduced into a clear and minimal set of diagnostic markers. Moreover, a measured path has been set forth to establish the extent that these markers have clinical utility in supporting prevention therapy paving the road for rational health management and development of insurance reimbursement programs. It is expected that this and similar approaches will lead to real personalization of care in AD as well as other medical conditions for the benefit of patients, care givers, and health systems.

### Diagnostics by adaptation of 3D convolutional networks for AD

**Figure 13:**
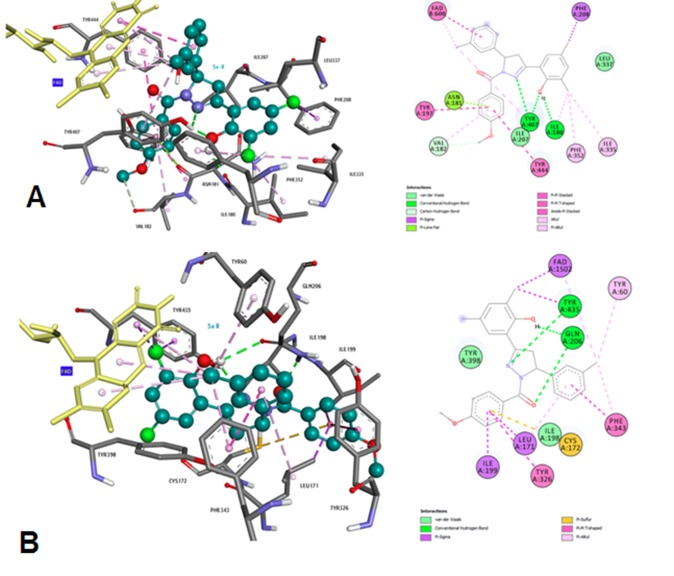
Pre-trained genetic features specified by fine-tuned task with image data are shown.

AD leads to the death of nerve cells and tissue loss throughout the brain. Thus, the treatment is to reduce the brain volume in size dramatically through time that is affecting its function. The estimated number of affected people will double for the next two decades so that one out of 85 persons will have AD by 2050. The necessity of having a computer-aided system for early and accurate AD diagnosis becomes critical as the cost of caring the AD patients is expected to rise dramatically. Several popular noninvasive neuro-imaging tools such as structural MRI (sMRI), functional MRI (fMRI), and positron emission tomography (PET) have been investigated for developing such a system. Multi-view features [154, 155, 156] from the available images are extracted using a classifier that trains to distinguish between different groups of subjects (AD, mild cognitive impairment (MCI), and normal control (NC)) groups. The sMRI has been recognized as a promising indicator of AD progression [157, 158, 159, 160, 161]. Various machine-learning techniques were employed to leverage multiview MRI, PET, and CSM data to predict AD. It was extracted from multi-view features using several selected templates from the MRI dataset of subjects. Tissue density maps of each template were used for clustering subjects within each class in order to extract an encoding feature for each subject. The use of support vector machine (SVM) to classify subjects is contextual. An implementation of the 3D-CNN [162, 163, 164, 165, 166, 167, 168] uses the ReLU activation functions at each inner layer and the fully connected upper layers with a softmax top-most output layer predicting the probability of belongs to an input brain sMRI to the AD, MCI, or NC group as shown in Figure 13. The Adadelta gradient descent was used to update the pre-trained 3D-CAE and to fine-tune the entire 3D-ACNN. The 3D-ACNN classifier can accurately predict AD on structural brain MRI scans than several other state-of-theart predictors. The pertaining and freezing layers were used to enhance feature generality in capturing the AD biomarkers. Moreover, three-stacked 3D CAE network were relevant on CAD Dementia dataset. The extracted learnt features (Table 2) are used for AD biomarkers detection in the bottom layers of 3D CNN network. Three fully connected layers are stacked on top of the bottom layers to form AD classification on 210 subjects in this network. The classification performance was measured using tenfold cross validation and compared to the state-of-the-art models. 3D CNN out-performed compared to other known methods.

### Prognostic factors for AD

The factors that influence the rate of functional and cognitive decline in AD are poorly understood. An investigation using geriatric inpatients and outpatients with a clinical diagnosis of AD based on DSM-III criteria were assessed with the Blessed Dementia Scale (BDS) and the Blessed Information-Memory- Concentration (BIMC) test at baseline and at 3, 6, and 12 months to identify prognostic factors. The rates of decline on both scoring systems varied widely among individuals are observed [169]. The only variable that significantly correlated with decline of functional status on the BDS was the initial cognitive score on the BIMC test; a higher BIMC score predicted a slow decline in function. Cognitive deterioration on the BIMC scale was faster in women than men and in younger than older patients, which confirms that the clinical course varies widely among patients with AD. It also shows that cognitive profiling at the onset of disease can help to predict disease progression and suggests that patients with early-onset of Alzheimer's may have more rapid cognitive deterioration [170]. In a slowly progressive disorder like AD, evaluation of the clinical effect for drug candidates requires large numbers of patients over extended treatment periods. Current cell- and animal-based disease models of AD are poor at predicting a positive treatment response in patients. The gap between disease models and large yet costly clinical trials with high failure rates has to be bridged where biomarkers for the intended biochemical drug effect may be of value. Such biomarkers are called 'theranostic' [171]. Therefore, it is of interest to review the literature addressing the prospective value of these biomarkers that evaluated the performance of novel Aβ isoforms as theranostic markers in AD from cell to patient [172].

### Nano medicine for the treatment of AD

There is no efficient therapy for AD but a promising approach is represented by nanotechnology, easily multi-functionalizable devices with size in the order of billionth of meter [173]. The development of nano-metric drug delivery systems permits a targeted and sustained release of old and new treatments offering a novel strategy to treat complex neuro-degenerative disorders [174]. Nano-based strategies for AD treatment aiming at carrying drugs across the blood-brain barrier (BBB) in particular to target the metabolism of Aβ peptide are promising. The theranostic nano-particles are built upon four basic components such as signal emitter, therapeutic payload, payload carrier, and targeting ligand. The signal emitter possesses certain unique optical, magnetic, or radioactive property, and can emit physical signals spontaneously or upon excitation by an external source. The signal can be detected by an external receiver and reconstructed to form images. The therapeutic payload can be chemotherapeutic drugs, or nucleic acids, such as DNA and siRNA. The payload carrier is generally a matrix commonly comprised of polymeric materials with multiple functional groups on which signal emitters or therapeutic payloads can be conjugated. The targeting ligand on the nano-particle is selected to bind to and form a complex with a specific disease marker on the target cell facilitating transport of theranostic nano-particle to the site of interest and enabling specific interactions with the target cell or tissue. The signal emitter and therapeutic payload of theranostic nano-particles can be either embedded in the carrier or conjugated on its surface while the targeting ligand is always covalently attached to the surface of the carrier, which allows the direct interaction with the target cell or tissue. Common multimodality nano-particle imaging agents include MRI-optical, MRIPET, and optical-PET agents [175, 176, 177, 178]. For example, iron oxide super paramagnetic nano-particles can be conjugated with a fluorophore to enable both MR and biophotonic imaging [169]. With this dual-imaging capability, MRI scans can be used to identify tumor localization for post-operation monitoring while biophotonic imaging with the resolution at the cellular level can be used intra-operatively to identify tumor boundaries for precise resection. Nano-particles have been used for the targeted delivery of drugs aiming to reduce the AD symptoms or to reverse the course of the disease [179, 180, 181, 182, 183]. The multi-valence of nanoparticles has allowed their functionalization with several kinds of targeting groups to cross the BBB and to target the place of treatment. With this approach an increased drug bioavailability has been achieved in the CNS using intravenous administration in place of more invasive administration routes. Nano-particles have also been used in the development of vaccines and therapeutic formulations for intranasal administration. Targeted nano-particles have been proved useful to enhance the performance of therapies against AD. A better understanding of AD mechanisms will help the successful application of targeted nano-particles for combined therapies.

## Conclusions

Computer aided drug discovery includes data mining, chemoinformatics, QSAR modeling, virtual screening, and molecular docking. We report a review on various computation methodologies used in CNS drug discovery processes such as the design of novel effective candidates for therapy of neurodegenerative AD. The use of sequential combination of ligands and structure-based virtual screening techniques with focus on pharmacophore models and molecular docking has been reported. The theragnosis (combination od diagnostic tests and therapy) paradigm for AD management involves using nanoscience to unite diagnostic and therapeutic applications to form a single agent or multiple functionalized pharmacies, allowing for diagnosis, drug delivery and treatment response monitoring. The application of this strategy to personalized AD care is envisioned.

## Conflict of interest

We have no conflicts of interest. This work received no specific grant from any funding agency in the public, commercial or notfor- profit sectors.

## Figures and Tables

**Table 1:** An update on selected anti-Alzheimer's disease drugs in clinical trials (updated in October 2017)

Target	Drug name	Therapy type	Trial status	Company
Serotoninergic(5-HT6)	Intepirdine	Small molecule	Phase II/III	Axovant Sciences Ltd.
Histaminergic(H3)	SUVN-G3031	Small molecule	Phase I	Suven Life Sciences Ltd
Glutaminergic	Riluzole	Small molecule	Phase II	Sanofi
BACE inhibitor	E2609	Small molecule	Phase III	Biogen, Eisai Co., Ltd.
	AZD3293	Small molecule	Phase III	AstraZeneca, Eli Lilly & Co.
	CNP520	Small molecule	Phase II/III	Amgen, Inc., Novartis Pharmaceuticals Corporation
	JNJ-54861911	Small molecule	Phase II/III	Janssen, Shionogi Pharma
	Verubecestat	Small molecule	Phase III	Merck
γ-Secretase inhibitor	NIC5-15	Small molecule	Phase II	Humanetics Pharmaceuticals Corporation
Aβ clearance	CAD106	Active Immunotherapy (Aβ1-6 peptides)	Phase II/III	Novartis Pharmaceuticals Corporation
	Gantenerumab	Passive immunotherapy (Against Aβ3-12 & Aβ18-27)	Phase III	Chugai Pharmaceutical Co., Ltd., Hoffmann-La Roche
Tau stabilization	TPI 287	Small molecule	Phase I	Cortice Biosciences
Tau aggregation inhibitor	TRx0237	Small molecule	Phase III	TauRx Therapeutics Ltd
p-Tau clearance	AADvac-1	Active immunotherapy (Synthetic peptide truncated and misfolded tau)	Phase II	Axon Neuroscience SE
Microglial activation inhibitor	Azeliragon	Small molecule	Phase III	Pfizer, TransTechPharma, Inc., vTv Therapeutics LLC
	CHF 5074	Small molecule	Phase II	CereSpir™ Incorporated, Chiesi Pharmaceuticals Inc.

**Table 2:** Effect of candidate SNPs for the conversion of mild cognitive impairment to Alzheimer's disease*

Gene	SNP	Chr.	Position	Minor/major2meta-analysis				AgeCoDe sample				DCN sample			ACE sample			ADC sample		
Allele	P-value	HR	σHR	I2	P-value	HR	σHR	P-value	HR	σHR	P-value	HR	σHR	P-value	HR	σHR
ABCA7	Rs3764650	19	1 046 520	G/T	0.24	0.9	0.08	0	0.83	0.96	0.17	0.31	0.72	0.23	0.6034	0.9	0.1	0.25	0.76	0.18
ADAMST20	Rs7295246	12	43 967 677	G/T	0.43	1.04	0.05	0	0.88	0.99	0.09	0.25	1.2	0.19	0.7416	1	0.1	0.45	1.11	0.15
BIN1	Rs7561528	2	127 889 637	A/G	0.55	1.03	0.06	0	0.76	0.97	0.1	0.19	1.25	0.22	0.9193	1	0.1	0.48	1.11	0.16
CASS4	Rs7274581	20	55 018 260	C/T	0.67	0.96	0.08	0	0.95	1.01	0.17	0.36	1.27	0.33	0.4805	0.9	0.1	0.46	0.82	0.21
CD2AP	Rs10948363	6	47 487 762	G/A	0.65	0.97	0.06	0	0.56	0.93	0.11	0.97	0.99	0.18	0.7333	1	0.1	0.82	1.04	0.16
CR1	Rs3818361	1	207 784 968	C/T	0.93	0.99	0.08	19	0.6	0.94	0.12	0.24	0.78	0.16	0.1693	1.2	0.1	0.67	0.93	0.16
ECHDC3	Rs7920721	10	11 720 308	G/A	0.43	1.04	0.05	0	0.59	1.06	0.11	0.5	0.89	0.15	0.5159	1.1	0.1	0.47	1.11	0.16
EPHA1	Rs10808026	7	143 099 133	A/C	0.75	0.98	0.06	0	0.77	0.97	0.11	0.93	0.98	0.21	0.994	1	0.1	0.73	0.94	0.17
FRMD4A	Rs17314229	10	14 016 159	T/C	0.75	1.04	0.11	0	0.63	0.91	0.18	0.97	0.99	0.3	0.9779	1	0.2	0.17	1.44	0.38
INPP5D	Rs35349669	2	234 068 476	T/C	0.91	1.01	0.07	30	0.53	1.06	0.1	0.24	0.82	0.14	0.5088	1	0.1	0.15	1.23	0.18
MEF2C	Rs190982	5	88 223 420	G/A	0.19	1.1	0.08	44	0.62	0.95	0.1	0.5	1.12	0.19	0.0018	1.3	0.1	0.71	1.06	0.15
MS4A	Rs4938933	11	60 034 429	C/T	0.31	0.93	0.06	27	0.67	1.04	0.11	0.65	1.08	0.18	0.023	0.8	0.1	0.42	0.89	0.13
MTHFD1L	Rs11754661	6	151 207 078	A/G	0.85	0.98	0.11	0	0.84	1.05	0.24	0.97	0.99	0.29	0.2979	0.8	0.1	0.26	1.38	0.4
NDUFAF6	Rs7818382	8	96 054 000	T/C	0.18	1.07	0.05	0	0.5	1.07	0.11	0.53	1.1	0.16	0.4346	1.1	0.1	0.52	1.09	0.15
NME8	Rs2718058	7	37 841 534	G/A	0.38	1.09	0.11	69	0.68	0.96	0.09	0.02	1.49	0.25	0.262	0.9	0.1	0.09	1.29	0.19
PICALM	Rs3851179	11	85 868 640	A/G	0.51	0.96	0.05	0	0.87	0.98	0.1	0.57	1.1	0.19	0.6135	1	0.1	0.27	0.85	0.13
PTK2B	Rs28834970	8	27 195 121	C/T	0.98	1	0.06	8.6	0.9	1.01	0.11	0.13	0.77	0.13	0.9222	1	0.1	0.31	1.16	0.17
SCIMP	Rs7225151	17	5 137 047	A/G	0.11	1.13	0.08	0	0.99	1	0.15	0.95	0.98	0.25	0.0813	1.2	0.1	0.34	1.23	0.27
SPPL2A	Rs8035452	15	51 040 798	C/T	0.61	0.97	0.06	27	0.78	1.03	0.1	0.21	1.23	0.2	0.1252	0.9	0.1	0.36	0.87	0.13
TOMM40	Rs2075650	19	45 395 619	G/A	1.19e-14	1.62	0.1	0	1.02e−04	1.56	0.18	0	1.67	0.29	1.53e−07	1.8	0.2	0	1.49	0.19
TREML2	Rs9381040	6	41 154 650	T/C	0.76	0.98	0.08	41	0.97	1	0.11	0.05	0.7	0.13	0.2735	1.1	0.1	0.79	0.96	0.14
*Note: HRs was calculated with uni-variate Cox proportional hazard model with adjustment for age and gender.Abbreviations: ACE -the Fundacio ACE from Barcelona - ADC, Amsterdam Dementia Cohort; AgeCoDe - German study on Aging, Cognition and Dementia in primary care patients; Chr - chromosome; DCN - German Dementia Competence Network; HR -hazardratio; σHR - hazard ratio standard deviation; I2 - heterogeneity index; SNP - single-nucleotide polymorphism.																				

**Figure 9:**
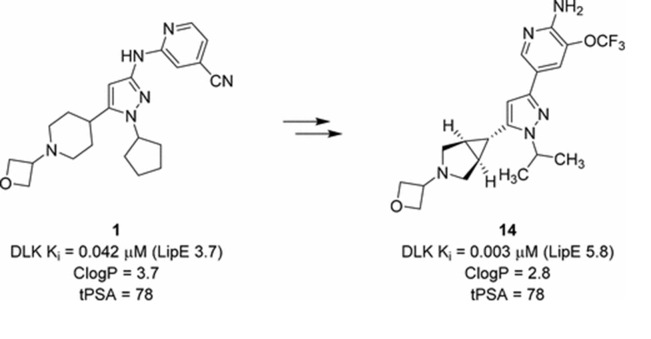
Selective inhibitors of Dual Leucine Zipper Kinase (DLK, MAP3K12) with known activity are shown in the context of AD.

**Figure 10:**
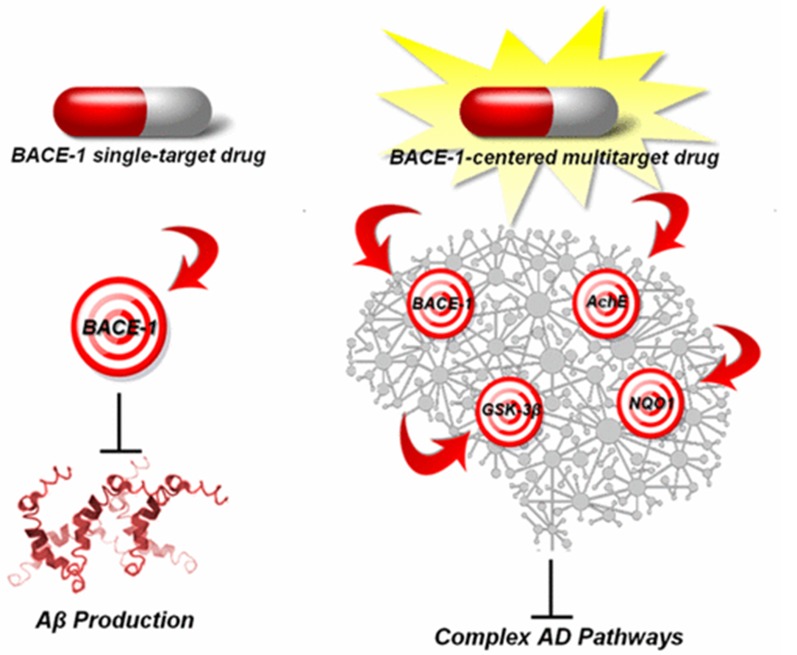
Paradigm shift from Single-Target Molecules to Multitarget Compounds for AD is shown using BACE-1 inhibitors.

## Abbreviations

AD: Alzheimer's disease
Aβ: amyloid beta
NFTs: neurofibrillary tangles
3D-CNN: 3Dimensional convolutional neural network
PD: Parkinson's disease
TNF- α inhibitor: Tumor Necrosis Factor - α inhibitor
APP: Amyloid precursor protein
MTDL: multi-target designed ligands
QSAR: quantitative structure-activity relationship
AChE: acetylcholinesterase
BuChE: butyrylcholinesterase
MAO: monoamine oxidase
NCATS: National Center for Advancing Translational Sciences
BACE1: beta-site APP cleaving enzyme1
ADME/T: absorption, distribution, metabolism, excretion/toxicity
sMRI: structural MRI
fMRI: functional MRI
PET: positron emission tomography
MRI+CLP: MRI with cleft lip and palate
MMSE: Mini Mental State Examinations
DLK: dualleucinezipperkinase
PDB: Protein Data Bank
CHARMM: Chemistry at Harvard Macromolecular Mechanics
FAD: flavin adenine dinucleotide
LOAD: late-onset Alzheimer's disease
TOMM40: Translocase of outer mitochondrial membrane
APOE: Apolipoprotein E
MCI: mild cognitive impairment
NC: normal control
SVM: support vector machine
ReLU: rectified linear unit
3D-CAE: 3D Computer-aided engineering
CADDementia: computer-aided diagnosis of dementia
ADNI: Alzheimer's Disease Neuroimaging Initiative
BDS: Blessed Dementia Scale
BIMC: Blessed Information-Memory-Concentration
DSM: The Diagnostic and Statistical Manual of Mental Disorders
ROS: reactive oxidative stress
CSM: Content storage management
CAE: Computer-aided engineering;
BBB: blood brain barrier
